# CS5931, a Novel Polypeptide in *Ciona savignyi*, Represses Angiogenesis via Inhibiting Vascular Endothelial Growth Factor (VEGF) and Matrix Metalloproteinases (MMPs)

**DOI:** 10.3390/md12031530

**Published:** 2014-03-13

**Authors:** Ge Liu, Ming Liu, Jianteng Wei, Haijuan Huang, Yuyan Zhang, Jin Zhao, Lin Xiao, Ning Wu, Lanhong Zheng, Xiukun Lin

**Affiliations:** 1 Institute of Oceanology, Chinese Academy of Sciences, 7 Nanhai Road, Qingdao 266071, China; E-Mails: liug878@163.com (G.L.); Wuning2283720@126.com (N.W.); 2 The University of Chinese Academy of Sciences, Beijing 100049, China; 3 Key Laboratory of Marine Drugs, Ministry of Education, School of Medicine and Pharmacy, Ocean University of China, Qingdao 266003, China; 4 Lanzhou Institute of Chemical Physics, Chinese Academy of Sciences, Lanzhou 730000, China; E-Mail: Weijt@163.com; 5 Qingdao University of Science and Technology, Qingdao 266042, China; E-Mails: qkhuanghaijuan@126.com (H.H.); zhangyuyanlcs@163.com (Y.Z.); 6 Department of Biotechnology, Zhengzhou University, Zhengzhou 450001, China; E-Mail: zhaojinwang2136@126.com; 7 Qingdao Agricultural University, Qingdao 266109, China; E-Mail: xiaolin_qd@163.com; 8 Yellow Sea Fisheries Research Institute, Chinese Academy of Fishery Sciences, Qingdao 266071, China; E-Mail: Zhenglanhong@126.com; 9 Department of Pharmacology, Capital Medical University, Beijing 100069, China

**Keywords:** CS5931, anti-angiogenesis, zebrafish, HUVECs, VEGF and MMPs

## Abstract

CS5931 is a novel polypeptide from *Ciona* *savignyi* with anticancer activities. Previous study in our laboratory has shown that CS5931 can induce cell death via mitochondrial apoptotic pathway. In the present study, we found that the polypeptide could inhibit angiogenesis both *in vitro* and *in vivo*. CS5931 inhibited the proliferation, migration and formation of capillary-like structures of HUVECs (Human Umbilical Vein Endothelial Cell) in a dose-dependent manner. Additionally, CS5931 repressed spontaneous angiogenesis of the zebrafish vessels. Further studies showed that CS5931 also blocked vascular endothelial growth factor (VEGF) production but without any effect on its mRNA expression. Moreover, CS5931 reduced the expression of matrix metalloproteinases (MMP-2 and MMP-9) both on protein and mRNA levels in HUVEC cells. We demonstrated that CS5931 possessed strong anti-angiogenic activity both *in vitro* and *in vivo*, possible via VEGF and MMPs. This study indicates that CS5931 has the potential to be developed as a novel therapeutic agent as an inhibitor of angiogenesis for the treatment of cancer.

## 1. Introduction

Angiogenesis, the sprouting or intussusception of pre-existing blood vessels to form new vessels, involves many coordinated endothelial cell activities, including proliferation, migration, alignment, and cord formation [1,2]. It is well documented that angiogenesis is a prerequisite of solid tumor growth, since the tumor progression and metastasis depend on the existence of a functional blood supply system [3,4,5,6]. The angiogenesis process was regulated by many angiogenic factors; VEGF (vascular endothelial growth factor) is one of the major regulators of angiogenesis and can stimulate the growth of blood vessels directly; MMPs (matrix metalloproteinases), particularly MMP-2 and MMP-9, involved in extracellular matrix degradation [7], are crucial for the endothelial cell migration, organization and hence, angiogenesis [8,9]. Therefore, VEGF and MMPs are closely related to the angiogenesis, and targeting these factors has become a promising anticancer strategy [6,10]. In recent years, a number of anti-angiogenic agents have progressed to clinical studies [7,11]. However, most of the anti-angiogenic candidates do not target tumor cells specifically and can cause endothelial dysfunction and vessel pruning in healthy tissues, and what is more, can induce drug resistance of cancer and stromal cells [12,13]. To overcome these defects, the continuous effort in searching for novel anti-angiogenic agents remains pressing. 

The ocean is an important source of biologically active substances and numerous novel peptides with anticancer activity have been isolated from marine organisms [14,15,16]. Many of them have been reported to display anti-angiogenic activity. For example, Somocystinamide A (ScA), a lipopeptide derived from *L.majuscula*, could inhibit angiogenesis by inducing apoptosis in angiogenic endothelial cells [17]. Aplidine, a cyclic depsipeptide isolated from the Mediterranean tunicate *Aplidium* *albicans*, also displayed anti-angiogenic activity both *in vitro* and *in vivo* [5]. 

In our previous study, a novel polypeptide, CS5931, with potent antitumor activity, was purified from *Ciona* *savignyi*. We found that CS5931 polypeptide displayed significant cytotoxicity against several cancer cells and induced apoptosis in HCT-8 cells via mitochondrial-mediated pathway [18]. Sequence analysis reveals that there is high homology between CS5931 and human granulin A in their primary and 3D structure [19]. However, little is known about its mode of action. Our primary study shows that CS5931 also inhibits the proliferation of HUVEC cells, suggesting the possibility of its anti-angiogenic activity. In the present study, we evaluated the effects of recombinant CS5931 polypeptide on proliferation, migration and cord formation of HUVECs, and the effect of the polypeptide on the formation of new vessels in zebrafish embryos was also examined. 

## 2. Results and Discussion

### 2.1. CS5931 Inhibits the Proliferation of HUVECs

**Figure 1:**
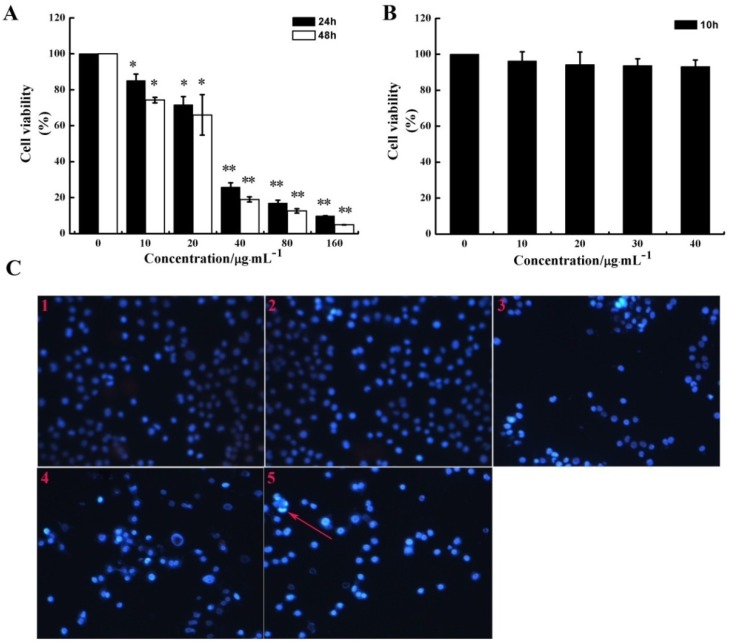
Effect of CS5931 on HUVEC proliferation. The cells were incubated in the absence or presence of certain concentrations of CS5931 at 37 °C for 24 h, 48 h (**A**) and 10 h (**B**) and cell viability was determined by MTT assay as described in the Materials and Methods section. (**C**) Cells were stained by PI/Hoechst 33258 after they were treated with 0 (1), 10 (2), 20 (3), 30 (4), 40 μg/mL (5) CS5931 for 24 h and pictures were taken using a fluorescence microscopy. The apoptotic cell with bright blue fluorescence was indicated by an arrow. Results are normalized to untreated cells. All experiments were repeated more than three times. Values represent the means ± SD of triplicate measurements. * *P* < 0.05, ** *P* < 0.01 *versus* medium control.

MTT assay was performed to determine if CS5931 could inhibit the proliferation of HUVECs. As shown in Figure 1A, the polypeptide caused a significant growth-inhibiting effect on HUVECs compared with the untreated HUVECs; the proliferation rate decreased by 74.3, 83.1 and 90.2% after incubation for 24 h, while it decreased by 81.0, 87.4 and 95.1% after incubation for 48 h, when treating the cells with CS5931 at a concentration of 40, 80 and 160 μg/mL, respectively. 

A previous study has shown that CS5931 induced cell death via mitochondrial-mediated pathway in human colon cancer HCT116 cells. In order to determine if the polypeptide can induce apoptosis in HUVEC, PI/Hoechst 33258 staining assay was performed. Our results showed that treatment with higher concentration of CS5931 for 24 h could induce endothelial cell apoptosis to some extent (Figure 1C). These results revealed that the repression of HUVEC growth induced by CS5931 is in a dose and time dependent manner (Figure 1A,B) and the polypeptide could inhibit the proliferation of HUVEC cells via apoptosis pathway when treating the cells for more than 24 h.

It is reported that some cytotoxic antitumor drugs can affect endothelial cell functions and angiogenesis [20,21]. However, not all of them are true anti-angiogenic agents because they need a higher drug concentration to achieve inhibitory effect in endothelial cells than that in tumor cells [22,23]. CS5931 inhibits endothelial cell proliferation, with an IC_50_ similar to that observed for tumor cells [19], indicating that the anti-angiogenic effect might indeed occur in tumors and contribute to the final anticancer activity.

**Figure 2:**
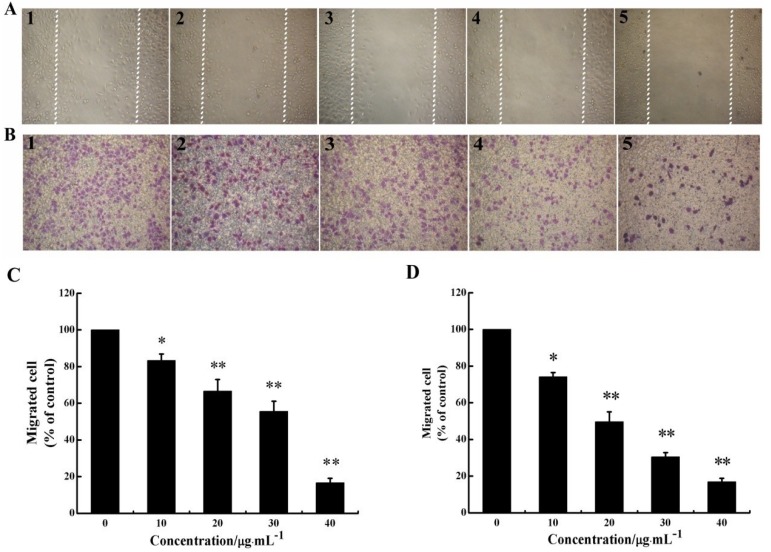
CS5931 inhibits the migration of HUVECs. Cells were treated without (1) or with 10 (2), 20 (3), 30 (4) and 40 μg/mL (5) of CS5931. After incubation for 8 h, cell migration was analyzed using scratch-wound assay (**A**) as well as Transwell assay (**B**). (**C**) Quantitative evaluations of HUVECs migration induced by CS5931 in the scratch-wound assay and Transwell assay (**D**). Results are normalized to untreated cells. All experiments were repeated more than three times. Values represent the means ± SD of triplicate measurements. * *P* < 0.05, ** *P* < 0.01 *versus* medium control.

### 2.2. CS5931 Represses the Migration of HUVECs

As migration of endothelial cell is necessary for angiogenesis, we investigated the effect of CS5931 on directional cell motility using a scratch-wound assay and Transwell assay. The results of scratch-wound assay showed that the wound healing was gradually reduced as the concentration of the polypeptide increased in a dose-dependent relationship (Figure 2A,C). The results of Transwell assay revealed that treatment with CS5931 resulted in a concentration-dependent suppression of cell migration; the inhibition rate of the cells was 25.8, 50.3, 69.4 and 83.1%, when treating the cells with CS5931 at a concentration of 10, 20, 30 and 40 μg/mL, respectively (Figure 2B,D). Both the findings indicated that CS5931 could prevent HUVECs migration significantly. Since the inhibition of cell migration by CS5931 occurred at exposure times at which cell proliferation was not obviously affected (Figure 1B) and cell apoptosis did not happen, the results suggested that CS5931 might indeed exert its anti-angiogenic effect by affecting HUVEC migration. 

### 2.3. CS5931 Disrupts the Cord Formation of HUVECs

**Figure 3:**
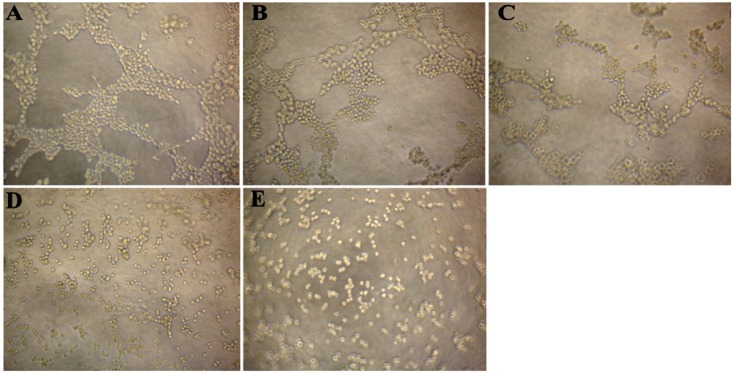
Effect of CS5931 on the formation of capillary-like structures of HUVECs. HUVECs were seeded on the surface of the Matrigel in a 96-well plate and treated without (**A**) or with 10 (**B**), 20 (**C**), 30 (**D**) and 40 μg/mL (**E**) of CS5931. After incubation for 6 h, capillary tube formation was examined using an inverted microscope.

Since tube formation is an important process in angiogenesis, we then explored the effects of CS5931 on capillary-like tube structure formation ability. Three-dimensional layer of Matrigel experiment was performed and the results showed that the capillary-like tube formation was inhibited significantly when treating the cells with CS5931 (Figure 3B–E); higher concentration of CS5931 abrogated the cord formation completely (Figure 3E). In contrast, the capillary-like tube structure network could be clearly found in cells untreated with the polypeptide (Figure 3A). These findings demonstrated that CS5931 could suppress endothelial cell cord formation. 

The antimotility activity of CS5931 was apparently sufficient to confer true anti-angiogenic activity. This was further supported by the finding that CS5931 prevented cord formation *in vitro*, an assay that mimics the final events during angiogenesis, when endothelial cells become aligned and organized in a capillary-like structure. Once again, the inhibitory effect occurred before endothelial cell proliferation was dramatically affected and apoptosis has not happened, suggesting that CS5931 might indeed prevent the process of angiogenesis.

### 2.4. CS5931 Blocks Vessel Formation in Zebrafish Embryos *in Vivo*

Zebrafish has been used as an ideal model to evaluate the angiogenic activity of anticancer agents. In the present study, SIV (subintestinal vessel) formation of zebrafish embryos was measured to assess the anti-angiogenic activity of CS5931 *in vivo*.

**Figure 4:**
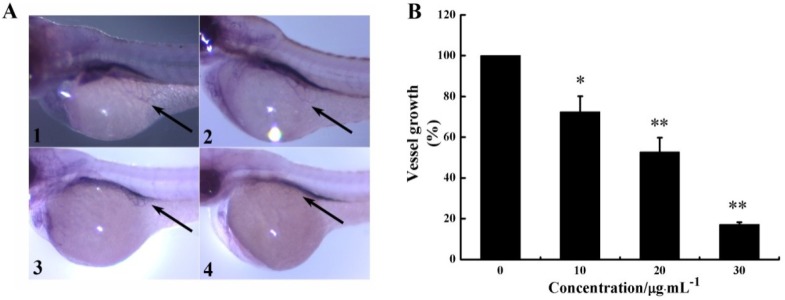
CS5931 affects subintestinal vessel (SIV) formation in zebrafish embryos. (**A**) Lateral view of stained zebrafish embryos at 72 hpf. Embryos were treated without (1), or with 10 (2), 20 (3), and 30 μg/mL (4) of CS5931. Vessels of zebrafish embryos were observed using a dissecting stereomicroscope. SIVs were indicated by an arrow. (**B**) Quantification of growth repression of SIVs induced by CS5931. Results are normalized to untreated embryos. All experiments were repeated more than three times. Values represent the means ± SD of triplicate measurements. * *P* < 0.05, ** *P* < 0.01 *versus* medium control.

As seen in Figure 4A, CS5931 prevented SIV formation in zebrafish embryos significantly; treatment with 10, 20, 30 μg/mL CS5931 reduced SIVs growth by 27.6, 47.2 and 82.8%, respectively (Figure 4B). Our results confirmed that CS5931 significantly inhibited angiogenesis both *in vitro* and *in vivo*. Cisplatin, a cytoxic antineoplastic drug, can inhibit endothelial cell proliferation *in vitro* but not affect angiogenesis *in vivo* [24,25,26,27], implying that drugs affecting endothelial cell proliferation are not necessarily anti-angiogenic. Our study revealed that the polypeptide also affected the growth of SIVs in zebrafish embryos. The results suggest that CS5931, unlike cisplatin, not only affects angiogenesis of HUVECs *in vitro*, but also impairs the development of angiogenesis *in vivo*. 

### 2.5. CS5931 Reduces VEGF Expression in HUVECs

**Figure 5:**
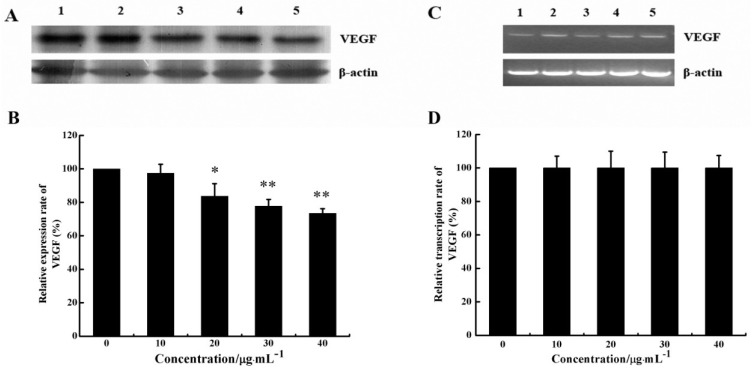
Effect of CS5931 on the expression of VEGF protein and mRNA. Cells were treated without (1) or with 10 (2), 20 (3), 30 (4) and 40 μg/mL (5) of CS5931, respectively. After incubation for 24 h, the VEGF expression was analyzed by Western Blotting (**A**) and RT-PCR analysis (**C**). (**B**) and (**D**) represent the quantitative evaluation of the expression of VEGF. Results are normalized to untreated cells. All experiments were repeated more than three times. Values represent the means ± SD of triplicate measurements. * *P* < 0.05, ** *P* < 0.01 *versus* medium control.

Since VEGF is one of the foremost regulators of angiogenesis, we investigated whether CS5931 could decrease VEGF expression of HUVECs both at mRNA and protein levels using RT-PCR and Western Blotting. As shown in Figure 5A,B, the VEGF protein level decreased significantly in cells treated with CS5931. To further investigate if CS5931 affected the expression of VEGF mRNA, we also assessed the VEGF mRNA expression in HUVECs. The results showed that after treatment of the cells with CS5931 for 24 h, the mRNA expression of VEGF remained basically unchanged (Figure 5C,D). These results suggested that the effect of CS5931 on VEGF expression occurs at the translational level. However, the exact mechanism of action still remains unknown; whether the polypeptide affects the translational efficiency of VEGF mRNA or increases its degradation still needs to be addressed. 

Several peptides from marine organisms have been identified with anti-angiogenic activity. Aplidine, a peptide isolated from the Mediterranean tunicate *Aplidium* *albicans*, displays potent anti-angiogenic activity [5] with an obvious inhibitory effect on VEGF production [28]. A polypeptide, PG155, isolated from Shark Cartilage in our laboratory possesses strong anti-angiogenic activity both *in vitro* and *in vivo* [29]; PG155 also suppresses the expression of VEGF in HUVECs. This study also reveals that CS5931 displays its anti-angiogenic activity via the repression of VEGF expression. 

### 2.6. CS5931 Down-Regulates the Secretion and mRNA Expression of MMPs

**Figure 6:**
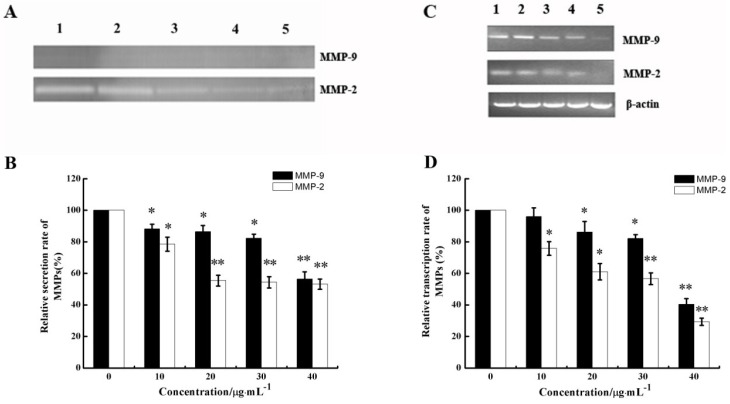
Effect of CS5931 on the secretion of matrix metalloproteinases (MMPs) protein and mRNA expression. HUVECs were treated without (1) or with 10 (2), 20 (3), 30 (4) and 40 μg/mL (5) of CS5931, respectively. After incubation for 24 h, the secretion of MMPs protein was analyzed by gelatin zymography (**A**) and MMPs mRNA expression was analyzed by RT-PCR (**C**). (**B**) and (**D**) represent the quantitative analysis of MMPs secretion and mRNA expression. Results are normalized to untreated cells. All experiments were repeated more than three times. Values represent the means ± SD of triplicate measurements. * *P* < 0.05, ** *P* < 0.01 *versus* medium control.

Given that the process of cell invasion requires enzymatic digestion of the matrix, we investigated whether CS5931 affected the production of matrix-degrading metalloproteinases in endothelial cells. Gelatin zymography was carried out to investigate whether CS5931 affects the gelatinolytic activity of MMP-2 and MMP-9 secreted from endothelial cells. As shown in Figure 6A, CS5931 suppressed MMP-2 and MMP-9 secretion in a dose-response pattern. The quantitative analysis of the gelatinolytic activity indicated that, when cells were treated with 10, 20, 30, and 40 μg/mL CS5931, MMP-2 activity was reduced by 21.6, 44.6, 45.6 and 46.8%, while MMP-9 activity was reduced by 11.9, 13.5, 17.8 and 43.6%, respectively (Figure 6B). These results indicated that CS5931 inhibited the secretion of MMP-2/9 in endothelial cells. 

The mRNA levels of MMP-2/9 were further examined by semi-quantitative RT-PCR after HUVECs were treated with certain concentrations of CS5931 for 24 h. As shown in Figure 6C, CS5931 could inhibit the gene expression of MMP-2/9 in a concentration-dependent manner. The inhibition rate of MMP-2 and MMP-9 was about 70.7% and 59.7%, respectively, when treating the cells with CS5931 at a concentration of 40 μg/mL for 24 h (Figure 6D). These results revealed that CS5931 not only inhibited the expression of MMP-2/9 mRNA, but also eliminated the secretion of MMP-2/9 and MMPs, which are also involved in the anti-angiogenic activity induced by CS5931. 

## 3. Experimental Section

### 3.1. Materials and Reagents

The recombinant CS5931 polypeptide was prepared in our laboratory according to the method described by Zhao *et al.* [19]. Transwell inserts with 8 µm pores polycarbonate filters was purchased from Corning company (Corning Costar, Cambridge, MA, USA). Matrigel was provided by BD company (Becton Dickinson, Bedford, MA, USA). All other reagents used in the experiment were analytical grade. 

### 3.2. Cells and Cell Culture

HUVECs were purchased from American Type Culture Collection and cultured at 37 °C in a humidified atmosphere of 5% CO_2_ and 95% air in RPMI-1640 (GIBCO, Invitrogen, Grand Island, NY, USA) supplemented with 10% fetal bovine serum (FBS; GIBCO, Invitrogen, Grand Island, NY, USA). All experiments were carried out with the same batch of HUVECs. 

### 3.3. HUVEC Proliferation Assay

The anti-proliferative effects of CS5931 on the growth of HUVECs were examined *in vitro* using MTT assay [30]. In brief, cells (3.5 × 10^3^/well) were plated in the 96-well plate in RPMI-1640 medium containing 10% FBS and cultured at 37 °C for 24 h. Then, CS5931 with certain concentrations were added to the medium. After incubation for 48 h, MTT solution (5 mg/mL, 20 µL) was added into each well and incubated for another 4 h. Then, 150 µL DMSO was added, and the plate was gently agitated until the color reaction was uniform and the OD490 was determined by a microplate reader with subtracted background absorbance. The inhibition ratio was calculated as follows:

Inhibition ratio (%) = (OD_control group_ − OD_experimental value_)/OD_control group_ × 100%

The IC_50_ value was expressed as the concentration of drugs causing 50% inhibition.

### 3.4. PI/Hoechst 33258 Staining Assay

HUVECs were counted and plated in 24-well plates and cultured at 37 °C for 24 h. CS5931 with certain concentrations (0, 10, 20, 30 and 40 μg/mL) were added to the medium. After incubation for another 24 h, cells were stained with propidium iodide (PI) and Hoechst 33258 and then examined by fluorescence microscopy.

### 3.5. Scratch-Wound Cell Migration Assay

HUVECs were counted and plated in 96-well plates. After growth to 90% confluence, “scratch” wounds were created by scraping cell monolayers with sterile disposable yellow tips [31,32,33]. After washing the scratched cell monolayers with PBS twice, RPMI-1640 medium supplemented with 2% FBS containing certain concentrations of CS5931 was added. After incubation for 10 h, 3 fields of each wound were selected and photographed with an inverted phase-contrast microscope. The number of HUVECs migrated from the edge of the injured monolayer was quantified by measuring the distance between the wound edges before and after injury. The migration rate at the start of the experiment was measured and arbitrarily defined as 100%. After incubation for 10 h, the width of the remaining wound was measured and the migration inhibition rate was calculated using the following formula:

Inhibition rate (%) = [1 − (average size in drug treated group/average size in control group)] × 100%

### 3.6. Transwell Migration Assay

Migration of HUVECs was determined in a Transwell Boyden chamber (Corning Costar, Cambridge, MA, USA) containing a polycarbonate filter with a pore size of 8 µm as described previously [29]. In brief, cell suspension (5 × 10^5^ cell/mL, 100 μL) containing certain concentrations of CS5931 (0, 10, 20, 30 and 40 μg/mL) was added to the upper compartment of each well. The lower compartment contained 0.6 mL of RPMI-1640 medium supplemented with 20% FBS. After incubation for 8 h at 37 °C, the filter was removed and fixed with 95% ethanol and stained with 0.1% crystal violet. HUVECs remained on the upper side of the filter were removed with the cotton swabs. Cells on the lower surface of the filter (migrated) were counted manually with a digital camera in five random fields and the average number of total fields was calculated. The inhibition rate of migration was calculated as using the following formula:

Inhibition rate = [1 − (migrated cells treated/migrated cells control)] × 100%

### 3.7. HUVEC Tube Formation Assay

The ability of HUVEC to form capillary-like structures on a 3D layer of basement membrane (Matrigel) was tested as described previously [26]. In brief, ice-cold Matrigel (BD, Bedford, MA, USA) diluted with serum-free RPMI-1640 medium was layered in a 96-well plate and incubated at 37 °C for 30 min to allow polymerisation. HUVECs (3 × 10^4^ cell/well) were plated onto the Matrigel layer in the culture medium containing certain concentrations of CS5931 (10, 20, 30 and 40 μg/mL) for 6 h. Photographs from randomly chosen fields were taken using a digital camera when cords were formed. 

### 3.8. Alkaline Phosphatase Staining for Visual Inspection in Zebrafish Embryos

Zebrafish embryos were generated by natural pairwise mating as described in the zebrafish handbook [34]. Embryos at 24 h post-fertilization (hpf) were manually dechorionated with 1 mg/mL trypsin for 10 min at room temperature immediately prior to drug treatment. Embryos treated with CS5931 were maintained in 24-well plates (20 embryos/well) for an additional 48 h. 

Vessel staining was performed as described previously [29,35,36]. Briefly, at 72 hpf, embryos were fixed with 4% paraformaldehyde for 2 h at room temperature, then washed three times in PBS and dehydrated by immersing in 25, 50, 75 and 100% methanol in PBT buffer and rehydrated stepwise to 100% PBT. The embryos were then equilibrated in NTMT buffer (0.1 M Tris-HCl at pH 9.5, 50 mM MgCl_2_, 0.1 M NaCl, and 0.1% Tween-20) at room temperature for 45 min. The staining reaction was started by incubating embryos with NBT/BCIP solution for about 45 min and the reaction was stopped by adding PBST. Embryos were then immersed in a solution of 5% formamide and 10% hydrogen peroxide in PBS for 30 min to remove endogenous melanin in the pigment cells and allow full visualization of the stained vessels. Embryos were then examined on a dissecting stereomicroscope (Zeiss, Jena, Germany). Images were collected and stored using digital camera and total SIV vessel length was determined by point-to-point measurement using Image J software.

### 3.9. Western Blotting Analysis

To determine the effect of CS5931 on VEGF protein, Western Blotting was performed as previously described [37]. Briefly, HUVECs were placed in a 6-well plate for 24 h, and CS5931 with certain concentrations (0, 10, 20, 30 and 40 μg/mL) was added and incubated for another 24 h. Cells were collected by trypsin digestion method and then lysed in the ice-cold RIPA lysis buffer supplemented with a protease inhibitor cocktail. The protein concentration of the lysates was quantified using the BCA protein assay (Pierce, Thermo Scientific, Waltham, MA, USA). Cell lysates (50 μg per lane) were resolved by 15% SDS-PAGE and transferred to nitrocellulose membranes. After incubation in blocking solution (7.5% non-fat milk) for 2 h, nitrocellulose membranes were incubated with 1:1000 dilution primary antibodies to human VEGF and β-actin proteins overnight at 4 °C. Membranes were incubated with 1:2000 dilution of HRP-conjugated second antibody for 1 h at room temperature and then the signal was detected with the enhanced chemiluminescence system and the relative intensity was analyzed using Image J software.

### 3.10. Production of Matrix Metalloproteinases (MMPs)

HUVEC cells were seeded in a 6-well plate for 24 h. CS5931 with certain concentrations were added. After incubation for another 24 h, the MMPs were analyzed using gelatin zymography assay as described previously [5,9,38]. Briefly, the presence of MMP-2/9 in the serum-free supernatants was analyzed by electrophoresis on an 8% non-denaturing SDS-PAGE containing 0.1% gelatin. After electrophoresis, the gel was washed with 2.5% Triton-X100 four times for 15 min to remove SDS and incubated in 50 mM Tris-HCl buffer, pH 7.6, containing 0.15 M NaCl, 10 mM CaCl_2_, and 0.02% (w/v) Brij-35 for 42 h at 37 °C. Gels were then stained with 0.5% Coomassie brilliant blue R250 in 25% methanol and 10% acetic acid, and destained in the same solution without Coomassie blue. Gelatinolytic activity was visualized by negative staining and the results were analyzed using gel documentation system and Image J software. 

### 3.11. Semi-Quantitative Reverse Transcription and Polymerase Chain Reaction (RT-PCR)

Total cellular RNA was isolated from HUVEC cells and RT-PCR was performed using PrimeScript RT reagent kit with gDNA Eraser (TaKaRa Bio Group, Otsu, Japan), according to manufacturer’s protocol. Briefly, cDNA was synthesized using total RNA (2 μg), Oligo dT Primer, and dNTP Mixture. The sequences of the primers used are shown in Table 1. 

### 3.12. Statistical Analysis

Data was expressed as mean ± standard deviation (SD). Statistics was analyzed using Student’s *t*-test. *P* values less than 0.05 were considered statistically significant.

**Table 1:** Primers.

	Primers	Sequences	Base pairs
P1	β-actin primer forward	5′-ACACTGTGCCCATCTAGGAGG -3′	21
P2	β-actin primer reverse	5′-AGGGGCCGGACTCGTCATACT-3′	21
P3	VEGF primer forward	5′-TTGCTGCTCTACCTCCAC-3′	18
P4	VEGF primer reverse	5′-AATGCTTTCTCCGCTCTG-3′	18
P5	MMP-2 primer forward	5′-GGCCCTGTCACTCCTGAGAT-3′	20
P6	MMP-2 primer reverse	5′-GGCATCCAGGTTATCGGGGA-3′	20
P7	MMP-9 primer forward	5′-CGGAGCACGGAGACGGGTAT-3′	20
P8	MMP-9 primer reverse	5′-TGAAGGGGAAGACGCACAGC-3′	20

PCR reactions were carried out in 50 μL of reaction mixture containing 25 μL Premix Taq DNA polymerase buffer, 0.5 μL of each primer (20 μM), and 2 μL cDNA. PCR was performed under the following conditions: an initial denaturation step at 94 °C for 5 min, followed by 35 cycles at 94 °C for 30 s, 60 °C for 30 s, 72 °C for 1 min, and a final extension step at 72 °C for 10 min. PCR fragments were separated on a 2% agarose gel. After ethidium bromide staining, the gels were illuminated at UV transilluminator and photographed and the relative intensity was analyzed using Image J software. 

## 4. Conclusions

Taken together, our study confirmed that the marine polypeptide CS5931 inhibits angiogenesis both *in vitro* and *in vivo*. VEGF and MMPs play an important role in the anti-angiogenic activity of the polypeptide. Considering its low molecular weight as well as its high production in genetic preparation, CS5931 possesses the potential to be developed as a novel anticancer agent. Further study is in progress in our laboratory to address if the polypeptide can inhibit tumor growth in xenograft nude mice model.
